# Reduced Cardiotoxicity of Ponatinib-Loaded PLGA-PEG-PLGA Nanoparticles in Zebrafish Xenograft Model

**DOI:** 10.3390/ma15113960

**Published:** 2022-06-02

**Authors:** Hissa F. Al-Thani, Samar Shurbaji, Zain Zaki Zakaria, Maram H. Hasan, Katerina Goracinova, Hesham M. Korashy, Huseyin C. Yalcin

**Affiliations:** 1 Biomedical Research Center, Qatar University, Doha P.O. Box 2713, Qatar; hissa.althani@qu.edu.qa (H.F.A.-T.); ss1104227@student.qu.edu.qa (S.S.); zain.zakaria@qu.edu.qa (Z.Z.Z.); mhasan@qu.edu.qa (M.H.H.); 2 Department of Biomedical Science, College of Health Sciences, QU Health, Qatar University, Doha P.O. Box 2713, Qatar; 3 Department of Pharmaceutical Sciences, College of Pharmacy, QU Health, Qatar University, Doha P.O. Box 2713, Qatar; kago@ff.ukim.edu.mk (K.G.); hkorashy@qu.edu.qa (H.M.K.); 4 Faculty of Pharmacy, Ss. Cyril and Methodius University in Skopje, Mother Theresa 47, 1000 Skopje, North Macedonia

**Keywords:** zebrafish, leukemia, nanomedicine, nanoparticle, pre-clinical, cardiotoxicity, cancer, Ponatinib, xenograft, PLGA

## Abstract

Tyrosine kinase inhibitors (TKIs) are the new generation of anti-cancer drugs with high potential against cancer cells’ proliferation and growth. However, TKIs are associated with severe cardiotoxicity, limiting their clinical value. One TKI that has been developed recently but not explored much is Ponatinib. The use of nanoparticles (NPs) as a better therapeutic agent to deliver anti-cancer drugs and reduce their cardiotoxicity has been recently considered. In this study, with the aim to reduce Ponatinib cardiotoxicity, Poly(D,L-lactide-co-glycolide)-b-poly(ethyleneoxide)-b-poly(D,L-lactide-co-glycolide) (PLGA-PEG-PLGA) triblock copolymer was used to synthesize Ponatinib in loaded PLGA-PEG-PLGA NPs for chronic myeloid leukemia (CML) treatment. In addition to physicochemical NPs characterization (NPs shape, size, size distribution, surface charge, dissolution rate, drug content, and efficacy of encapsulation) the efficacy and safety of these drug-delivery systems were assessed in vivo using zebrafish. Zebrafish are a powerful animal model for investigating the cardiotoxicity associated with anti-cancer drugs such as TKIs, to determine the optimum concentration of smart NPs with the least side effects, and to generate a xenograft model of several cancer types. Therefore, the cardiotoxicity of unloaded and drug-loaded PLGA-PEG-PLGA NPs was studied using the zebrafish model by measuring the survival rate and cardiac function parameters, and therapeutic concentration for in vivo efficacy studies was optimized in an in vivo setting. Further, the efficacy of drug-loaded PLGA-PEG-PLGA NPs was tested on the zebrafish cancer xenograft model, in which human myelogenous leukemia cell line K562 was transplanted into zebrafish embryos. Our results demonstrated that the Ponatinib-loaded PLGA-PEG-PLGA NPs at a concentration of 0.001 mg/mL are non-toxic/non-cardio-toxic in the studied zebrafish xenograft model.

## 1. Introduction

Cancer is the second leading cause of death worldwide with a high number of incidents [1]. Cancer evolves from mutations that cause the activation of oncogenes or/and inactivation of the tumor suppressor genes leading to uncontrolled cell growth and proliferation, which further trigger other complications in the body that eventually might lead to death [2]. Leukemia is a type of cancer that is characterized by the uncontrolled growth of the hematopoietic stem cells from the bone marrow [3]. There are several subtypes of leukemia and the most encountered subtype among adults is CML [3]. CML is generally diagnosed by the presence of the Philadelphia chromosome that harbors the *BCR-ABL* oncogene, which would cause abnormal cell proliferation and complications in the patients [4].

Therefore, the demand for successful anti-cancer therapeutics and the development of effective tools for early cancer detection and screening have increased. For example, the introduction of TKIs [5] such as imatinib, nilotinib, Ponatinib, and dasatinib as anti-cancer drugs particularly for CML has aided in improving the overall outcomes of the patients and increasing their survival rates [6,7]. However, due to some encountered toxicity of these drugs, especially to the heart [8], the necessity to employ nanotechnology in anti-cancer treatments has increasingly progressed [9,10]. This is due to the higher efficiency and precision of nanoparticles (NPs) in targeting cancer cells and reducing toxicity associated with anti-cancer drugs [11].

Due to the biocompatibility and biodegradability of the PLGA and PEG triblock copolymers, they have been extensively used in drug delivery [12,13]. Moreover, PLGA and PEG polymers have gained the approval of the US Food and Drug Administration (FDA) and the European Medicine Agency (EMA) to be utilized in many therapeutic applications due to their low toxicities and solubilization effects [14,15]. Moreover, PEG polymer can prolong the half-life and the circulation period of the NPs in the body by permitting further reticuloendothelial system recognition. This is because PEG polymers have steric stability and they can selectively evade the attachment of the opsonin proteins on the NPs surface [12,16]. Furthermore, the PLGA and PEG triblock copolymer has shown their ability to deliver several different anti-cancer drugs such as cisplatin, methotrexate, doxorubicin [17], and irinotecan [18].

Zebrafish have been used as a research model in many applications, such as cancer and neuronal disorders studies, due to their numerous unique characteristics [19,20,21]. For example, they have a high genetic resemblance to humans with about 70% orthologue genes, making them a useful model for genetic manipulation [22]. Moreover, they are easy to maintain, have short maturation and developing time, and their transparent embryos have made imaging and studying internal organs such as the heart much easier [23]. In addition, due to their lack of adaptive immunity during the first months of development, zebrafish are a good model for the xenotransplantation of human tumor cells to develop a cancer model to study human cancers and testing of anti-cancer drugs [24]. Zebrafish are also considered a useful animal model for investigating and screening the toxicity of several agents such as anti-cancer drugs [25].

In this study, we first developed and characterized PLGA-PEG-PLGA NPs and then loaded the generated PLGA-PEG-PLGA NPs with different concentrations of Ponatinib. Next, we tested these NPs efficacy in reducing the cardiotoxic effect of Ponatinib in the zebrafish xenograft model. According to our results, PLGA-PEG-PLGA NPs demonstrated their efficacy in reducing the well-known cardiotoxic side effects of Ponatinib in our studied model.

## 2. Materials and Methods

### 2.1. Cell Culture

Human CML K-562 cell line was obtained from the American-type culture collection (ATCC) (ATCC, Manassas, VA, USA) and as a kind gift from Shahab Uddin Khan from the interim Translational Research Institute (iTRI) at Hamad Medical Corporation (HMC), Doha, Qatar. Cells have been cultured according to the optimum conditions described by the manufacturer. The cells were cultured in RPMI 1640 supplemented with 10% FBS, 10,000 U/mL Penicillin-Streptomycin, and 100× GlutaMAX at 37 °C in a humidified 5% CO2 incubator. All reagents were obtained from Gibco (ThermoScientific, Waltham, MA, USA). The cells’ culture medium was changed every other day to obtain the optimum cell count and maintain their viability at 90% following this equation: No. of viable cells/total No. of cells × 100. The cell counting was performed by obtaining all the cell suspension from the T75 flasks into sterile (15 mL or 50 mL) tubes, then centrifuged at 1300 rpm for 5 min using centrifuge 5804 (Eppendorf, Hamburg, Germany), the supernatant was discarded and then the pellet was re-suspended in 3–2 mL RPMI 1640 media. Then the cell count was performed manually using a KOVA™ Glasstic™ Slide 10 with Grids (Fisher Scientific, Waltham, MA, USA) by taking 20 μL of the cell suspension mixed with 20 μL of the trypan blue stain (ThermoScientific, Waltham, MA, USA) and then 20 μL of the mixture was loaded in the hemocytometer. Only the cells in the large 4 squares at the edges were counted under a light microscope. After that, the cell count in an ml was done following the equation: cell count × dilution factor (2) × the hemocytometer constant (10^4^). After that to determine how much media were required to add into each T-75 flask for passaging the cells the following was followed:

$$\frac{\mathrm{No}.\;\mathrm{of}\;\mathrm{cell}\;\mathrm{count}\;\times \;\mathrm{how}\;\mathrm{much}\;\mathrm{media}\;\mathrm{was}\;\mathrm{added}\;\mathrm{to}\;\mathrm{the}\;\mathrm{pellet}\;\times 2}{8\times 10^{5}}$$

### 2.2. Fluorescent Labeling of CML Cells before Xenotransplantation

Once the K-562 cells have reached confluency (1 × 10^6^ cells/mL), they have been harvested by pelleting using a centrifuge at 1200 rpm for 5 min, the supernatant was then discarded then re-suspended in 3 mL PBS mixed with 6 μL of 5 μg/mL CM-Dil fluorescent dye Invitrogen (ThermoScientific, Waltham, MA, USA). Then, the labeled cells were incubated for 5 min at 37 °C followed by a 15–20 min incubation at 4 °C. After that, the cells were checked under the fluorescence microscope Olympus IX73 (Olympus, Hamburg, Germany) using fluorescent filters with excitation/emission spectra of 553/570 nm maxima.

### 2.3. Zebrafish Husbandry

Wild-type zebrafish embryos (AB strain) were used for this experiment. All animal experiments were carried out according to national and international guidelines for the use of zebrafish in experimental settings [26] and following the animal protocol guidelines required by Qatar University and the policy on zebrafish research established by the department of research in the Ministry of Public Health, Qatar (Ministry of Public Health, 2017). This study has been approved by the Institutional Animal Care and Use Committee (IACUC) (QU-IACUC 019/2020).

### 2.4. Observation of Embryos

Zebrafish embryos were counted and investigated for survival and morphological changes in 24 h intervals for 3 days using Zeiss SteREO Discovery V8 microscope with a Hamamatsu Orca Flash high-speed camera and images were analyzed using HCImage software. Dead embryos were scored according to the opaque color which they exhibit. Observed abnormalities have been investigated and recorded. Non-viable embryos were eliminated as soon as they were observed, whereas embryos with abnormal development were kept till the endpoint of the experiment.

After the incubation period, 72 hpf, six embryos from each experimental group were stabilized using 3% methylcellulose and visualized under the microscope. A 10 s bright field video of the beating heart and the body was recorded for each embryo at 100 frames per second (fps). The same region in the dorsal aorta (DA) and the posterior cardinal vein (PCV) was localized to measure the flow velocity, arterial pulse, and vessel diameter using Viewpoints MicroZebralab version 3.6 application [20,21]. 

### 2.5. Xenograft’s Injection Procedure

The zebrafish embryos were exposed to Pronase for 10 min at 24 h post-fertilization to remove the chorion. After that, they were incubated till 2 or 3 days-post-fertilization (dpf) at 28 °C. Dechorionated embryos were transferred to an injection slide, and they were anesthetized with 1% Tricane solution (Western Chemical Inc, Ferndale, WA, USA) for destabilization. The fluorescently labeled K562 cells were then injected into the yolk sac to allow the cells to enter the blood circulation using a fashioned glass capillary needle (World Precision Instruments, Florida, FL, USA). About 300 K562 cells were injected into each embryo, using the Harvard Apparatus PLI 90A picolitre injector (Harvard Apparatus, Holliston, MA, USA) at the BRC zebrafish facility. The embryos were first anesthetized with 200 mg/L Tricane for 5 min and were aligned properly to have their body on one side to allow easier access to their yolk sacs. Then, a capillary needle was prepared using borosilicate glass microcapillaries following the setting on the Narishige PC-100 puller. A total of 10 µL of the cells’ solution was then loaded into the needle and the needle was placed into a manipulator. The manipulator was then adjusted until holding the needle at a 45° angle to an embryo. The needle tip was broken slightly with tweezers, and the cells’ solution was gently injected into the zebrafish embryos’ yolk sacs. After that, the xenotransplanted embryos were transferred into new plates and fresh egg water and kept at 34 °C till the endpoint at 7 dpf. The zebrafish larvae were imaged under the fluorescence microscope using the ZEISS ZEN Microscope software (Carl-Zeiss, GmbH, Munich, Germany) each day after injection to check the cancer cell spread and to measure the tumor size.

### 2.6. Preparation of the NPs

The Poly(D,L-lactide-co-glycolide)-b-poly(ethylene oxide)-b-poly(D,L-lactide-co-glycolide) PLGA- PEG- PLGA polymer used to generate the NPs was purchased from Akina Inc., USA. A total of 25 mg of PLGA- PEG- PLGA polymer (Mw 6000:10,000:6000), along with 5 mg of the fluorescently labeled (DLLA: GA 50:50; Mn 10,000–20,000, Sigma-Aldrich, St. Louis, MO, USA) were dissolved in 10 mL of Tetrahydrofuran (THF) (VWR, USA). To induce nanoprecipitation and embryonic NPs formation, the organic solution was transferred drop-by-drop into 20 mL Milli Q water containing 5 mg Pluronic F127 (Sartorius, Germany). The dispersion was kept overnight with a magnetic stirrer to evaporate the organic solvent. The next day, the NPs dispersion was filtered through a 0.45 micron filter, and the filtrate was placed in the ultrafiltration tube (Vivaspin^®®^ 20 Ultrafiltration Unit) (Sartorius Stedim Biotech, Germany) to wash and concentrate the NPs (three washing cycles with Milli Q water at 4500 RPM for 10 min).

Samples of drug-loaded NPs with increasing concentrations of the active substance were prepared using the previously described procedure for unloaded NPs, with the addition of 5 mg, 10 mg, and 15 mg Ponatinib in the organic solution. Moreover, three washing cycles were performed to remove the free unencapsulated drug and the excess of the surface agent and concentrate the NPs. NPs dispersions with known concentrations were prepared by redispersion of the concentrated NPs in a certain volume of Milli Q water.

### 2.7. NPs Characterization

#### 2.7.1. Transmission Electron Microscope (TEM)

TF20: Tecnai G2 200kV TEM (FEI, Hillsboro, OR, USA) has been used to characterize the PLGA-PEG-PLGA NPs. The procedure was carried out by the Central Laboratories Unit (CLU) at Qatar University, by depositing a large droplet (around 10 μL) from each NPs sample onto a TF20 holder, and images were then obtained using a voltage of 200 kV.

#### 2.7.2. Scanning Electron Microscope (SEM)

The particles’ surface morphology was assessed using NOVA NANOSEM 450 (N-SEM) (FEI, Hillsboro, OR, USA) by the Central Laboratories Unit (CLU) at Qatar University. SEM uses a field emission gun as a source of electrons. The electron beam then travels through the column while being adjusted by different lenses till reaching the sample. The electrons interact with the sample producing secondary electrons and characteristic X-rays that can be detected by a special detector to produce electron images and elemental spectra correspondingly.

#### 2.7.3. Nanoparticles’ Size

The size of PLGA-PEG-PLGA NPs has been measured by Zetasizer Nano ZS (Malvern Instruments, Malvern, UK). The cuvette was filled with the NPs dispersion and inserted into the machine after selecting the corresponding refractive index of the NP.

#### 2.7.4. Zeta Potential Measurement

The surface charges of the loaded and unloaded PLGA-PEG-PLGA NPs were determined by the Zetasizer Nano ZS (Malvern Instruments, UK). The machine measures the Zeta potential by using electrophoretic light scattering. The PLGA-PEG-PLGA refractive index was obtained from the literature [27] and the NPs solution was then placed in a disposable folded capillary cell to be processed by the machine.

#### 2.7.5. Ponatinib Dissolution Rate

To determine the dissolution rate of the loaded drug in the NPs, a dialysis membrane method was performed. This was performed using the Float-A-Lyzer G2 membrane (MWCO 20 kDa), which traps the particles inside and allows the loaded drug to be released into the surrounding media. The NPs solution of 1 or 0.5 mL has been loaded inside the dialysis tube which was placed inside a beaker filled with PBS buffer (pH 7.4) with a magnetic stirrer at 37 °C for 24 h. After that, samples were taken for HPLC analysis from the same spot of the PBS buffer at regular intervals (1 h, 3 h, 5 h, and 24 h) results are shown in (Supplementary Figure S6).

#### 2.7.6. High-Performance Liquid Chromatography (HPLC)

For the efficacy of and quantitative assessment of loading and drug content of encapsulated Ponatinib, the HPLC method was used. Adequate volume of Ponatinib loaded PLGA-PEG-PLGA NP dispersion was diluted with a mobile phase and the quantification procedure was performed as described below. The efficacy of encapsulation was determined using the following equation:

EE (%) = Amount of active substance in NP/Total amount of active substance × 100

Degree content was calculated using the equation:

DC (%) = Amount of active substance in NP/Total amount of NPs × 100

Quantitative analysis was performed on WATERS ACQUITY UPLC system HPLC system, using a C18 column, the flow rate of 1.2 mL/min, and injection volume of 5 μL. The mobile phase was composed of: (A) KH_2_PO_4_ 0.0037 mol/L (40%), pH 3.5 adjusted by H_3_PO_4_; and: (B) Acetonitrile (60%). The quantification was performed using PDA/UV detector at 250 nm wavelength.

### 2.8. Unloaded NPs Toxicity

The zebrafish embryos at 24 h post-fertilization (hpf) were exposed to 200 µL Pronase solution (1 mg/mL) (Sigma-Aldrich, MO, USA) for 10 min to remove the chorion. Dechorioned embryos were then evaluated under the stereomicroscope (Zeiss, GmbH, Germany) and segregated into 6-wells plates equally (about 20 or 24 embryos in each well). After that, different concentrations of the unloaded NPs were prepared to determine the optimum concentration that will not cause any toxicity to the zebrafish embryos. Different concentrations of NPs have been prepared by diluting the proper amount of the NPs in (0.3 mg/mL) 1-phenyl-2-thiourea (PTU) in the egg water, the concentrations were: 1, 0.75, 0.5, 0.25, 0.1 mg/mL. The embryos then were incubated with different concentrations of NPs at 30 °C and the survival rate was then measured at 48 and 72 hpf.

### 2.9. Loaded NPs Toxicity

The zebrafish embryos at 24 h post-fertilization (hpf) were exposed to 200 µL Pronase (1 mg/mL) solution for 10 min to remove the chorion. Dechorionated embryos were then evaluated under the stereomicroscope and segregated into 6-well plates equally (about 20 or 24 embryos in each well). After that, three different concentrations of loaded NPs with Ponatinib (5 mg, 10 mg, and 15 mg) were prepared to select the least toxic concentration for the zebrafish embryos. Different concentrations of NPs in PTU containing egg water were prepared as follows: 1, 0.75, 0.5, 0.25, 0.1, 0.05, 0.01, 0.005, 0.0025 mg/mL to choose for loading the Ponatinib drug. The negative control group was untreated zebrafish embryos kept in egg water. The embryos were then incubated at 30 °C and the survival rate was measured at 48 and 72 hpf. The survival rate was calculated by dividing the number of viable embryos by the total number of embryos multiplied by 100.

### 2.10. Xenograft Exposure to Loaded PLGA-PEG-PLGA NPs Assay

Injected 2-dpf zebrafish embryos were allowed to recover for half an hour after injecting of K562 cells before exposing them to 0.001 mg/mL loaded PLGA-PEG-PLGA NPs with 10 mg and 15 mg Ponatinib. The embryos were separated and placed into 6-well plates: two wells for each group (control, 10 mg, and 15 mg) with 10 embryos in each. The 0.001 mg/mL concentration of loaded PLGA-PEG-PLGA NPs was prepared by diluting it in egg water. For a total volume of 3 mL, the required amount for one well, i.e., 1.440 µL of the 15 and 10 mg NPs were diluted in egg water. Then the embryos were incubated at 34 °C and started to be imaged at 4-dpf.

### 2.11. Survival Rate Analysis

On day 2 pf, the dead embryos were removed from the 6-well cell culture plates to avoid influencing the surviving embryos during the toxicity experiments. The numbers of the dead, surviving, and abnormal embryos of each NPs concentration group were recorded until 3 dpf. The survival rate was calculated by dividing the number of viable embryos by the total number of embryos, multiplied by 100.

### 2.12. Cardiovascular Structure/Function Analysis

To assess the cardiovascular toxicity side effects of both unloaded and loaded NPs, the analysis was carried out at 3-dpf for the embryos in all the treated groups to see the influence of interference on cardiac function, structure, and blood flow. The treated embryos were first placed in a concave slide for imaging using 3% methylcellulose for immobilization. Under the Hamamatsu Orca high-speed camera and Zeiss Lumar V12 stereomicroscope (Carl-Zeiss, GmbH, Germany), images and high-speed time-lapse movies were recorded at about 100 fps for the heart and tail of each embryo through the HCImage software (Hamamatsu, Japan). Then to assess for heart failure due to the toxicity of the NPs, tail videos have been analyzed for the Red Blood Cells (RBCs) movement within the blood flow using the MicroZebraLab (Viewpoint, Lyon, France). Tracking the RBCs aids in measuring the blood velocity by following an in-house algorithm from Viewpoint for tracking RBCs. This algorithm has also been used to measure heart rate in beats per minute. Heartbeat and blood flow velocity parameters are widely used to assess cardiac function in zebrafish. Lower heartbeat and/or blood flow velocity indicates deteriorated heart function.

### 2.13. Statistical Analysis

Statistical analysis was performed using GraphPad Prism version 9.0.0 software (GraphPad Software, San Diego, CA, USA). Data were analyzed using one way-ANOVA with Dunnet’s multiple comparison test. A *p*-value of less than 0.05 was considered statistically significant. One asterisk (*) indicates *p* < 0.05, two asterisks (**) indicate *p* < 0.01, three asterisks (***) *p* < 0.001, and four asterisks (****) indicate *p* < 0.0001.

## 3. Results

### 3.1. Fluorescent K562

Olympus fluorescent microscope was used to image the fluorescent K562 CML cells stained with CM-Dil fluorescent dye. The mCherry fluorescent filter with excitation/emission spectra of 587/610 has been chosen to examine the fluorescent K562 CML cells as the CM-Dil fluorescent dye has an excitation/emission of 553/570 nm maxima. Supplementary Figure S2 represents an image of the fluorescent K562 cells at 60× magnification. As seen from the figure, most of the K562 cells were successfully fluorescently stained with CM-Dil dye.

### 3.2. PLGA-PEG-PLGA NPs Preparation and Characterization

#### 3.2.1. PLGA-PEG-PLGA NPs Preparation

In the course of the nanoprecipitation process, particles are generated by simultaneous polymer/drug nucleation, molecular growth, and aggregation during the micromixing of water and the organic solvent phase. Supersaturation is the force behind all these processes influencing the size and distribution of the NP population. The growth of the nanoparticles will terminate due to the combined effect of the polymer/drug dilution and steric hindrance of Pluronic F127 which deposits at the polymer core/water interface, affecting the aggregation dynamic, particle size, and the drug content of the NPs. Therefore, during the nanoprecipitation process, the type and ratio of solvent to non-solvent, as well as the polymer/drug/stabilizing agent concentrations, have to be carefully selected to achieve high efficacy of drug encapsulation, adequate particle size, and low polydispersity index [28,29,30]. Our preliminary experiments pointed to the 5 mg Pluronic F 127/20 mL water phase, among the tested 2.5, 5, and 10 mg Pluronic F127/20 mL, as the most favorable concentration leading to the highest drug loading. No significant improvement in drug loading or influence on targeted particle size and distribution with further increase of concentration from 5 to 10 mg was noticed. The addition of surfactant in the organic phase increased drug loading, however, and the particle size and particle size distribution were also significantly increased. Two types of organic solvents, THF, with lower density and surface tension, and DMSO showing higher density and surface tension compared to water were also tested. The improved micromixing of the water phase with the lower density and lower surface tension organic solvent contributed to the generation of high-uniformity batches with significantly smaller NPs without any compromise on the drug-loading efficacy. Further, polymer concentration was adjusted to 25 mg or 30 mg/10mL THF to avoid the slow-down effect on the micro-mixing due to increasing viscosity of higher concentrations of the polymer solution which might lead to increased particle size and polydispersity index. Finally, three increasing concentrations, 5 mg, 10 mg, and 15 mg Ponatinib in the polymer solution were also selected to test the influence of drug concentration on the efficacy of loading, particle size, and distribution. Samples with increasing concentration of Ponatinib showed improved efficacy of loading and drug content as well as acceptable particle size for passive tumor targeting. Considering the results presented above, the final selected formula from our preliminary design studies was 30 mg PLGA-PEG-PLGA polymer with increasing concentrations of Ponatinib (1:6, 1:3, and 1:2 drug/polymer ratio) and 5 mg Pluronic F125 in 20 mL of water to prepare formulation A, B, and C for further physicochemical, morphological, in vitro, and in vivo characterization.

#### 3.2.2. PLGA-PEG-PLGA NPs Morphology

Transmission and Scanning Electron microscopes (TEM and SEM), respectively, were used to characterize the shape of the PLGA-PEG-PLGA NPs. Supplementary Figure S3A represents the shape of PLGA-PEG-PLGA NP, by TEM micrograph and it shows the NPs with their characteristic round shape. Supplementary Figure S3B represents the shape of PLGA-PEG-PLGA NPs using SEM showing the 3D spherical shape of the NPs.

#### 3.2.3. PLGA-PEG-PLGA NPs Size

The size of loaded PLGA-PEG-PLGA NPs prepared with 5 mg, 10 mg, and 15 mg Ponatinib has been measured in water dispersion using the Zetasizer Nano ZS (Malvern Instruments, UK). The particle size range of the NPs with increasing loading of Ponatinib was from 80 to 100 nm. The Z-average hydrodynamic diameter (Dh (nm) ± SD, n = 6) of each NPs’ group was as follows: 74.55 nm ± 28.74 for the PLGA-PEG-PLGA NPs loaded with 5 mg Ponatinib (Supplementary Figure S4A), 125 nm ± 26.91 for the PLGA-PEG-PLGA NPs loaded with 10 mg Ponatinib (Supplementary Figure S4B) and 116.9 nm ± 42.92 for PLGA-PEG-PLGA NPs loaded with 15 mg Ponatinib (Supplementary Figure S4C).

The Z-average hydrodynamic diameter (Dh (nm) ± SD) of the unloaded PLGA-PEG-PLGA NPs was 84.33 nm ± 13.83 (Supplementary Figure S5), indicating that PLGA-PEG-PLGA NPs size increased with the loading of Ponatinib.

#### 3.2.4. PLGA-PEG-PLGA NPs Surface Charge

The surface charge of the PLGA-PEG-PLGA NPs (water dispersion) had been assessed to characterize more of its material properties; thus, its interaction properties with the biological system can be predicted. For that, the Zeta potential for the loaded PLGA-PEG-PLGA NPs had been measured by the Zetasizer Nano ZS (Malvern Instruments, UK), and the surface charge ((mV) ± SD, n = 5) of the particles showed a net positive charge of 12.3 mV ± 5.5; 15.2 mV ± 3.4 and 16.7 mV ± 2.5 for 15, 10 and 5 mg Ponatinib loaded PLGA-PEG-PLGA NPs.

The Zeta potential for the unloaded PLGA-PEG-PLGA NPs demonstrated a net negative surface charge with an average of −2.66 ± 0.185 (mV) ± STD Table 1. The surface charge of the Ponatinib is positive (protonation of its terminal methylpiperazinyl nitrogen) with an average of 30.86 mV+/−2.744 (n = 5) Table 2, indicating that the positive zeta potential of loaded NPs is an additional confirmation for successful drug loading into the drug-delivery system.

#### 3.2.5. Ponatinib Dissolution Rate from PLGA-PEG-PLGA NPs

Dissolution rate experiments were performed using the dialysis method. In a phosphate buffer pH 7.4 pointed to a very slow dissolution rate from the prepared Ponatinib PLGA-PEG-PLGA NPs with no burst release except for the (sample A) prepared using 5 mg Ponatinib or 1:6 drug/polymer ratio (25% of the drug was released within the first 3 hours). For samples B and C, prepared using 10 and 15 mg Ponatinib (1:3 and 1:2 drug/polymer ratio), respectively, less than 10% released drug was determined within 24 h from all the samples (HPLC analysis). Results are demonstrated in Supplementary Figure S6. These release pattern favors the accumulation of the drug at the site of action incorporated within the NPs at the same time decreasing the off-site effects and toxicity.

#### 3.2.6. Efficacy of Loading and Drug Content

The efficacy of loading and drug content increased with the increasing concentration of Ponatinib during preparation. Calculated values were 18 ± 3.3% (n = 6), 20.8 ± 2.1% (n = 6) and 21.8 ± 2.7% (n = 6) for 5 mg, 10 mg, and 15 mg Ponatinib; or 1:6; 1:3, and 1:2 drug to polymer ratio during the preparation of the NPs. Calculated drug content was 3%, 6.5%, and 10.5% for samples prepared with 1:6, 1:3, and 1:2 drug-to-polymer ratios, respectively.

### 3.3. Unloaded PLGA-PEG-PLGA NPs Toxicity

#### 3.3.1. Survival Rate

**Figure 1:**
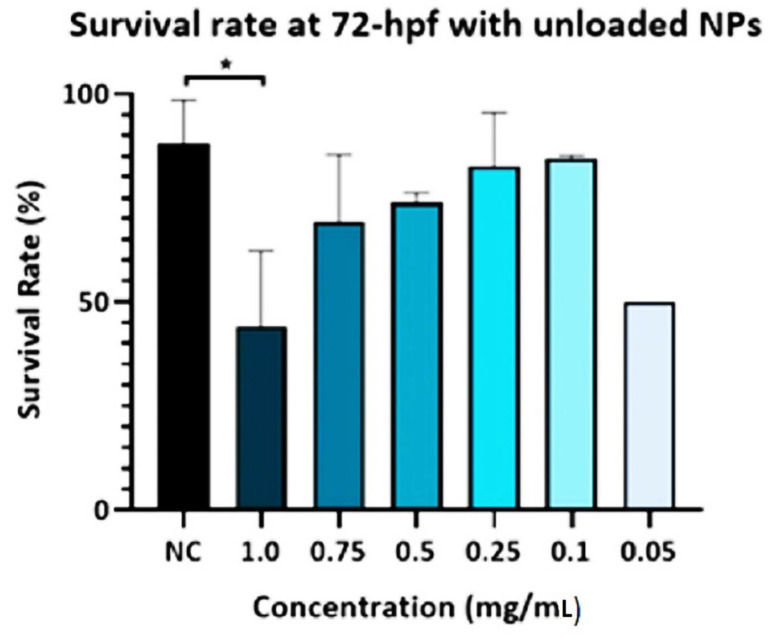
Survival rate of zebrafish embryos exposed to unloaded PLGA-PEG-PLGA NPs. The survival rate of the zebrafish embryos at 72 h post-fertilizing (hpf) was calculated for the negative control (NC) and the treated groups of different concentrations of unloaded PLGA-PEG-PLGA NPs. The survival rate of embryos exposed to unloaded PLGA-PEG-PLGA NPs (1 mg/mL) significantly decreased the survival rate of embryos compared to the NC, at 72 hpf. * = *p* < 0.05.

The survival rate of the zebrafish embryos at 72 h post-fertilizing (hpf) was calculated for the negative control (NC) which was untreated embryos kept in egg water and the treated groups of unloaded PLGA-PEG-PLGA NPs. Figure 1 indicates that there was a significant decrease in the survival rate of the 1.0 mg/mL group when compared to the negative control group. Meanwhile, the experimental groups with the lowest concentrations (0.75, 0.5, 0.25, 0.1, and 0.05 mg/mL) of unloaded PLGA-PEG-PLGA NPs did not show any significant difference when compared to the control group.

#### 3.3.2. Cardiac Function Assessment

**Figure 2:**
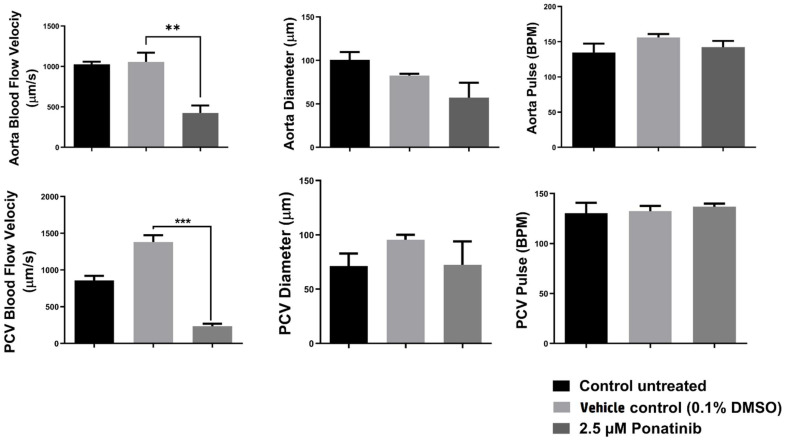
Cardiac function assessment and blood flow analysis for Ponatinib-tested embryos. Measurement of cardiac function for treated groups with Ponatinib at 72 hpf in comparison to the control untreated group (embryos were kept in egg water only) and the vehicle control group (0.1% DMSO in egg water); DMSO is the vehicle in which the Ponatinib drug was dissolved. All data are presented as mean ± SEM (6 embryos were used in each group; the experiment was performed in triplicate). D’Agostino and Pearson omnibus normality test was performed on all data to determine distribution. All parameters passed the test. Accordingly, a one-way-ANOVA with Sidak posthoc test was performed to compare pair: control vs. negative control and negative control and 2.5 μM Ponatinib. (**) = *p* < 0.01, (***) = *p* < 0.001.

First, we had investigated the effect of treating 72 hpf zebrafish embryos with Ponatinib (2.5 µm) on different cardiac function parameters. Cardiac function parameters measurements were examined on the zebrafish embryos’ two main blood arteries—(DA) dorsal aorta and (PCV) posterior cardinal vein—to examine the effect of Ponatinib treatment on the cardiovascular system of the zebrafish. Velocity, diameter, and pulse were measured using ZebraLab software. Cardiac parameters measurement was only possible on Ponatinib (2.5 μM) exposed embryos because of the severe effect of higher concentrations of Ponatinib on embryos’ viability. As demonstrated in Figure 2, Ponatinib reduced blood flow velocity (almost no flow) in both the DA and the PCV. Other tested parameters did not show a significant difference when compared to both the control untreated group, (embryos were kept in egg water only) and the vehicle control (embryos were exposed to 0.1% DMSO in egg water). Our findings showed that high Ponatinib concentrations may alter cardiomyogenesis in zebrafish.

**Figure 3:**
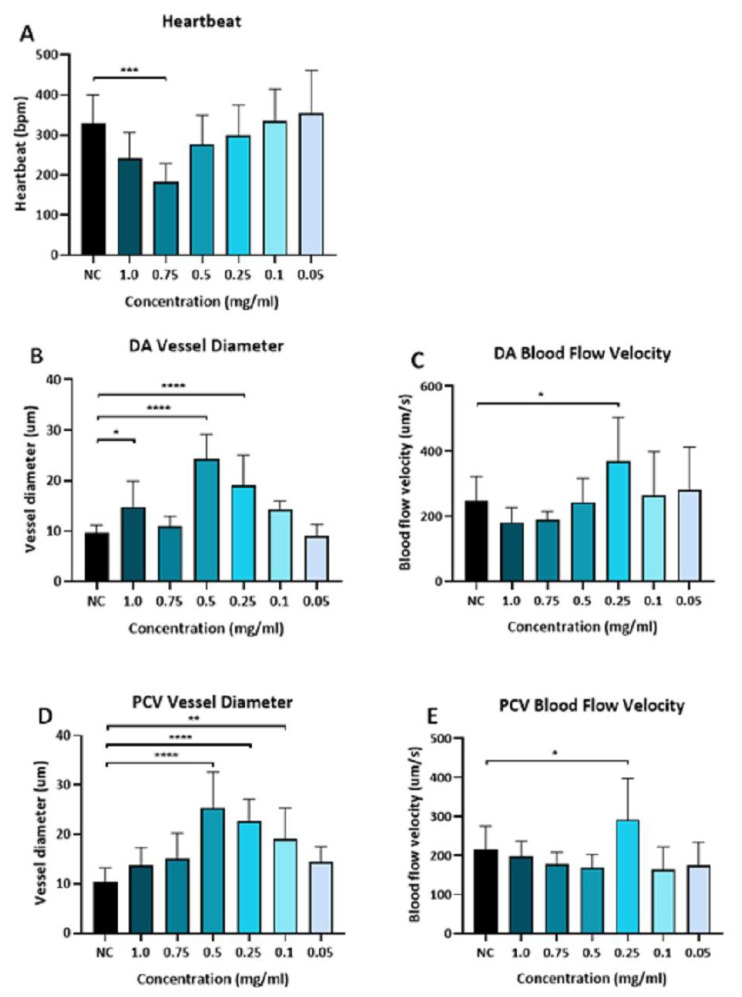
Cardiac function assessment of unloaded PLGA-PEG-PLGA. Cardiac function assessment of heart heartbeat, the dorsal aorta (DA) and posterior cardinal vein (PCV) vessel diameter, and blood flow velocity of the zebrafish embryos at 72hr post-fertilizing (hpf) treated with (0.75, 0.5, 0.25, 0.1, and 0.05 mg/mL) unloaded PLGA-PEG-PLGA NPs. (**A**) Heartbeat of embryos exposed to different concentrations of unloaded PLGA-PEG-PLGA NPs, a significant reduction in the heartbeat of the group which was treated with unloaded PLGA-PEG-PLGA NPs (0.75 mg/mL). (**B**) DA vessel diameter of embryos exposed to different concentrations of unloaded PLGA-PEG-PLGA NPs, vessel diameter showed to be enlarged significantly in groups of (1.0, 0.5, and 0.25 mg/mL) (**C**) DA blood flow velocity of embryos exposed to different concentrations of unloaded PLGA-PEG-PLGA NPs, blood velocity was increased significantly in the unloaded PLGA-PEG-PLGA NPs (0.25 mg/mL) group. (**D**) PCV vessel diameter of embryos exposed to different concentrations of unloaded PLGA-PEG-PLGA NPs, the vessel diameter showed to be enlarged in groups treated with unloaded PLGA-PEG-PLGA NPs (0.5, 0.25, 0.1 mg/mL). (**E**) PCV blood flow velocity of embryos exposed to different concentrations of unloaded PLGA-PEG-PLGA NPs, the blood velocity showed to be increased significantly in the unloaded PLGA-PEG-PLGA NPs (0.25 mg/mL) group. * = *p* < 0.05, (**) = *p* < 0.01, (***) = *p* < 0.001, (****) = *p* < 0.0001.

We followed this with the investigation of (DA) and (PCV) vessel diameter, and blood flow velocity in the zebrafish embryos at 72 h -post fertilizing (hpf) treated with (0.75, 0.5, 0.25, 0.1, and 0.05 mg/mL) unloaded PLGA-PEG-PLGA NPs. It was demonstrated that there was a significant reduction in the heartbeat of the group which was treated with unloaded PLGA-PEG-PLGA NPs (0.75 mg/mL) when compared to the negative control Figure 3A.

The dorsal aorta (DA) vessel diameter was seen to be enlarged in groups of (1.0, 0.5, and 0.25 mg/mL) and the blood velocity was increased significantly in the unloaded PLGA-PEG-PLGA NPs (0.25 mg/mL) group compared to the negative control Figure 3B,C.

In the posterior cardinal vein (PCV) the vessel diameter showed to be enlarged in groups treated with unloaded PLGA-PEG-PLGA NPs (0.5, 0.25, 0.1 mg/mL), and also the blood velocity increased significantly in the unloaded PLGA-PEG-PLGA NPs (0.25 mg/mL) group Figure 3D,E. Based on these results, we concluded that only a high concentration of unloaded NPs seems to be toxic to the animals.

### 3.4. Mortality and Visible Morphological Changes in Zebrafish

**Figure 4:**
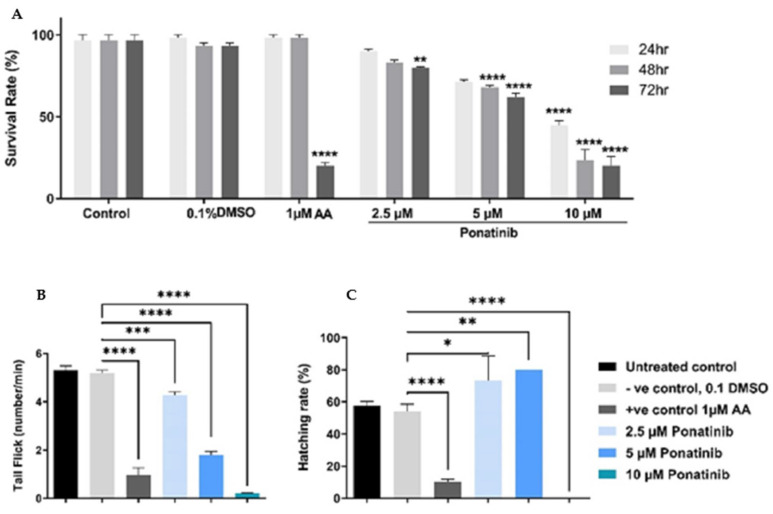
Mortality and visible morphological changes in Ponatinib-treated zebrafish embryos. (**A**) The survival rate of embryos exposed to different concentrations of Ponatinib compared to the Positive control (Aristolochic acid -AA) and NC (0.1% DMSO), at different timepoints: 24, 48, and 72 hpf, *n* = 20. (**B**) Assessment of potential neuro/muscular toxicity at 24 hpf by locomotion/tail-coiling assay. The plot represents the average tail coiling (burst/min) measured by DanioScope software. *n* = 20. (**C**) Ponatinib effect on the zebrafish embryos hatching rate *n* = 20. D’Agostino and Pearson omnibus normality test was performed on all data to determine distribution. All parameters passed the test. Accordingly, a one-way-ANOVA with Sidak posthoc test was performed to compare pair: Control vs. negative control and negative control and 2.5 mm Ponatinib. * = *p* < 0.05, (**) = *p* < 0.01, (***) = *p* < 0.001, (****) = *p* < 0.0001.

A simple visual comparison of treated zebrafish embryos to controls at 24, 48, and 72 hpf was undertaken to explore Ponatinib’s teratogenic potential. Aristolochic acid (AA) (1 µM) was used as a positive control (PC) that induces cardiac failure per prior research [31,32]. Ponatinib treatment drastically reduces embryos’ survival and tail flicking in a concentration-dependent manner, as demonstrated in Figure 4 panels A and B, respectively. The hatching rate of 48 h-treated zebrafish embryos was significantly different from that of untreated embryos. At low concentrations, Ponatinib increased the hatching rate significantly, while decreasing the hatching of embryos at a greater concentration (10 µM). At 72 hpf, survival was significantly reduced in the PC-treated group (AA, 1 µM).

### 3.5. Loaded PLGA-PEG-PLGA NPs Toxicity

#### 3.5.1. Survival Rate

**Figure 5:**
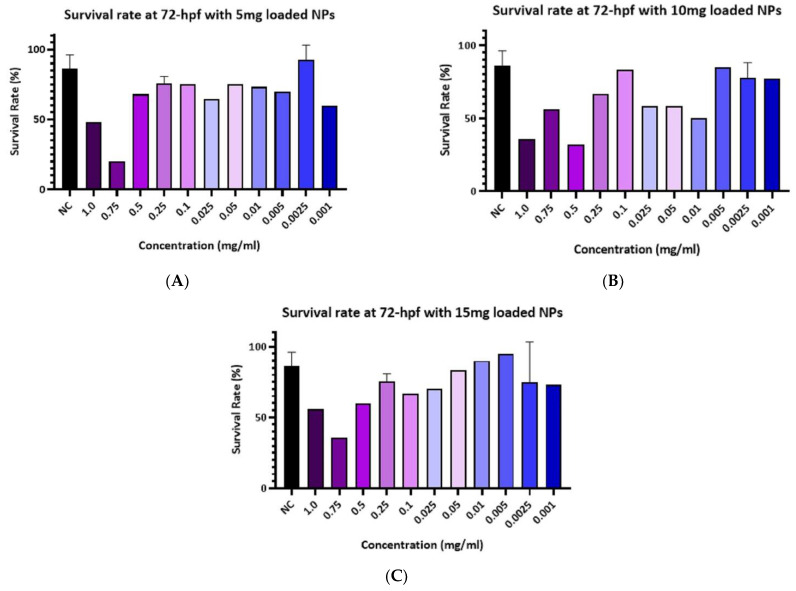
The survival rate of embryos exposed to different concentrations of Ponatinib-loaded PLGA-PEG-PLGA NPs. (**A**) The survival rate of embryos exposed to Ponatinib (5 mg) PLGA-PEG-PLGA NPs compared to the NC, at 72 hpf, higher concentrations of the loaded PLGA-PEG-PLGA NPs (1 and 0.75 mg/mL) had the lowest survival rate compared to the other groups. (**B**) The survival rate of embryos exposed to Ponatinib (10 mg) PLGA-PEG-PLGA NPs compared to the NC, at 72 hpf, groups that were treated with loaded PLGA-PEG-PLGA NPs (1 and 0.5 mg/mL) showed the lowest survival rate. (**C**) The survival rate of embryos exposed to different concentrations of Ponatinib (15 mg) loaded PLGA-PEG-PLGA NPs compared to the NC, at 72 hpf. The treated groups with loaded PLGA-PEG-PLGA NPs (1 and 0.75 mg/mL) showed the lowest survival rate when compared to the other groups.

The survival rate of the zebrafish embryos at 72 h post-fertilizing (hpf) was calculated for the negative control and the treated groups with three concentrations of loaded PLGA-PEG-PLGA NPs with Ponatinib: 3% of the drug content (sample A), 6.5% of drug content (sample B), and 10.5% of drug content (sample C). Figure 5A demonstrates the survival rate of treated embryos with different concentrations of the PLGA-PEG-PLGA NPs of Sample A. Data indicate that higher concentrations of the loaded PLGA-PEG-PLGA NPs (1 and 0.75 mg/mL) had the lowest survival rate compared to the other groups. Figure 5B demonstrates the survival rate of treated embryos with different concentrations of the PLGA-PEG-PLGA NPs loaded in sample B. Data indicate groups that were treated with loaded PLGA-PEG-PLGA NPs (1 and 0.5 mg/mL) showed the lowest survival rate. In Figure 5C, data demonstrates the survival rate of treated embryos with different concentrations of the PLGA-PEG-PLGA NPs of sample C, and it is indicated that the treated groups with loaded PLGA-PEG-PLGA NPs (1 and 0.75 mg/mL) showed the lowest survival rate when compared to the other groups.

Based on these results, only high concentrations of loaded NPs were toxic to the embryos and the concentration of PLGA-PEG-PLGA (0.001 mg/mL) of samples (B and C) showed a similar survival rate to the negative control. Thus, this concentration is considered to be the optimum for performing the next experiments.

#### 3.5.2. Cardiac Function Assessment

The cardiac function was assessed by analyzing the heartbeat, the dorsal aorta (DA) and posterior cardinal vein (PCV) vessel diameter, and blood flow velocity.

**Figure 6:**
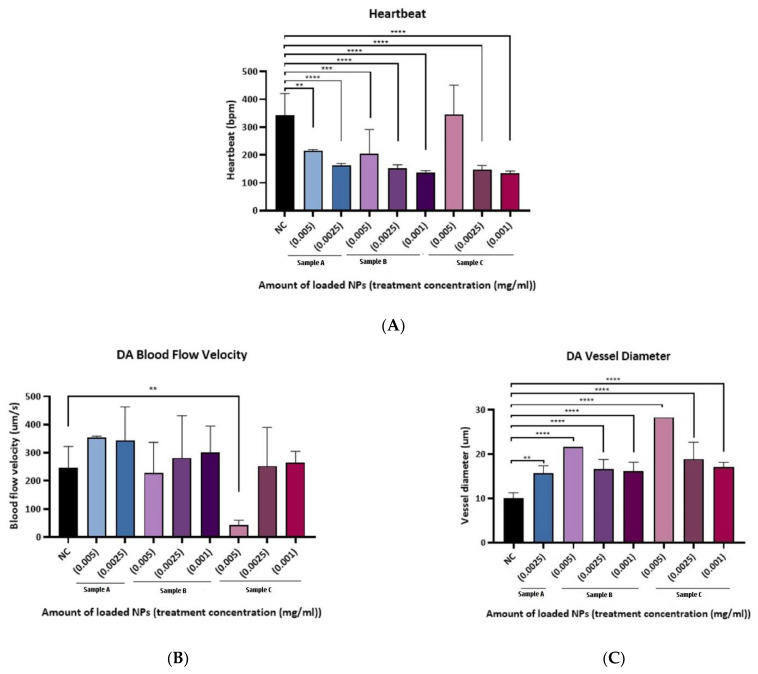

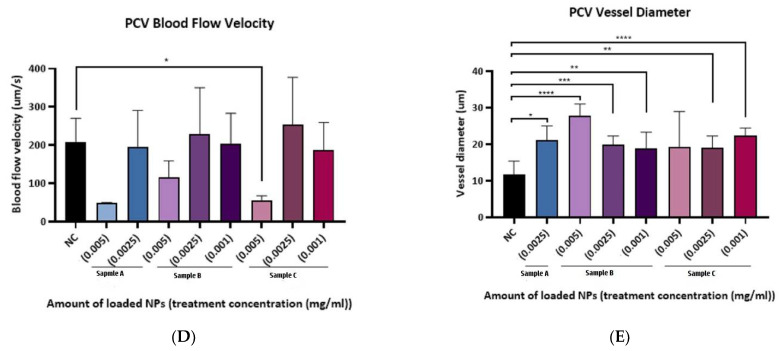
Cardiac function assessment of loaded PLGA-PEG-PLGA NPs. (**A**) Heartbeats of groups treated with sample A NPs (0.005 and 0.0025 mg/mL), sample B NPs (0.005, 0.0025, and 0.001 mg/mL), and sample C NPs (0.0025 and 0.001 mg/mL) were significantly reduced compared to (NC) (**B**) DA blood-flow velocity, blood velocity was significantly reduced in treated groups with sample C NPs in the concentration of (0.005 mg/mL) (**C**) The DA vessel diameter, an enlargement in the groups treated with sample A NPs at a concentration of (0.005 and 0.0025 mg/mL), sample B NPs at a concentration of (0.0025 mg/mL) and sample C NPs at a concentration of (0.0025 and 0.001 mg/mL) (**D**) PCV blood flow velocity was significantly reduced in the group treated with sample C NPs (0.005 mg/mL), and (**E**) PCV vessel diameter of embryos exposed to different concentrations of samples A, B, and C PLGA-PEG-PLGA NPs, was significantly enlarged in all treated groups when compared to control. * = *p* < 0.05, (**) = *p* < 0.01, (***) = *p* < 0.001, (****) = *p* < 0.0001.

The heartbeat of groups treated with sample A NPs (0.005 and 0.0025 mg/mL), sample B NPs (0.005, 0.0025 and 0.001 mg/mL) sample C NPs (0.0025 and 0.001 mg/mL) were significantly reduced compared to the negative control, as shown in Figure 6A.

Blood velocity was significantly reduced in treated groups with sample C NPs at a concentration of (0.005 mg/mL) and slightly high in the treated group with sample C NPs at a concentration of (0.0025 mg/mL). The dorsal aorta (DA) vessel diameter demonstrated an enlargement in the groups treated with sample A NPs at a concentration of (0.005 and 0.0025 mg/mL), sample B NPs at a concentration of (0.0025 mg/mL), and sample C NPs at a concentration of (0.0025 and 0.001 mg/mL) as shown in Figure 6B,C.

In the posterior cardinal vein (PCV), the measured vessel diameters were significantly enlarged in all drug and NPs combinations, except for the group treated with sample C NPs (0.005 mg/mL), where the blood velocity was significantly reduced in Figure 6D,E.

Relying on these data, the concentration of NPs (0.001 mg/mL) of samples B and C manifested a non-toxic effect on treated groups; therefore, these concentrations would be used further in the xenograft experiments.

### 3.6. Zebrafish Xenograft Model

**Figure 7:**
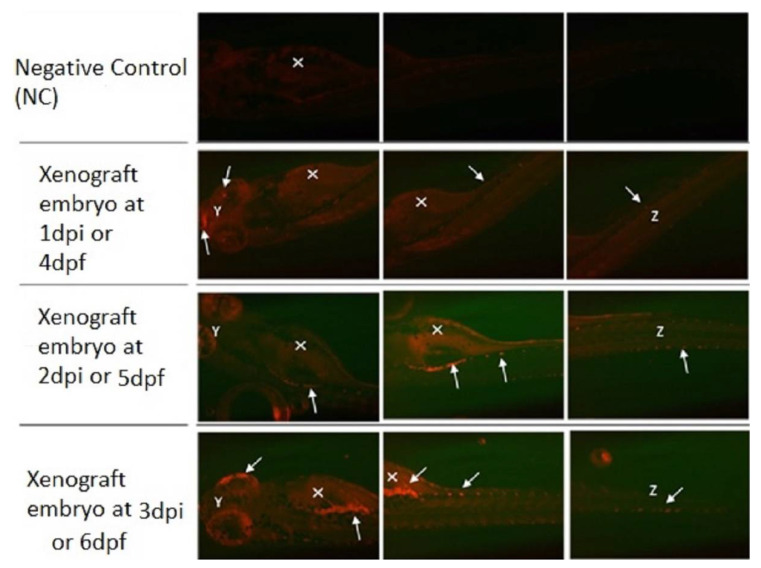
Zebrafish Xenograft model injected at 3 dpf. Representative fluorescence images of zebrafish screening at 4 dpf to 6 dpf using fluorescent microscopy and investigation of fluorescent K562 cells proliferation (white solid arrows) all over the animal body (Y—eyes; X—yolk sac; Z—tail) using mCherry fluorescence filter. Original magnification 100×.

K562 CML cell line was successfully transplanted into the 72 hpf zebrafish embryos. Figure 7 represents a xenografted embryo from 1 day-post-injection to 3 days-post-injection (dpi) compared to a negative control embryo to differentiate between the autofluorescence of the embryos. The fluorescently labeled cancer cells using CM-Dil red fluorescent dye demonstrated an increase in proliferation leading to an increase in the tumor size and the migration of cancer cells to distant sites of the embryo over time as indicated by the white arrows. The yolk sac area (white X), which was the injection site of the cells, showed to contain concentrated tumor cells.

**Figure 8:**
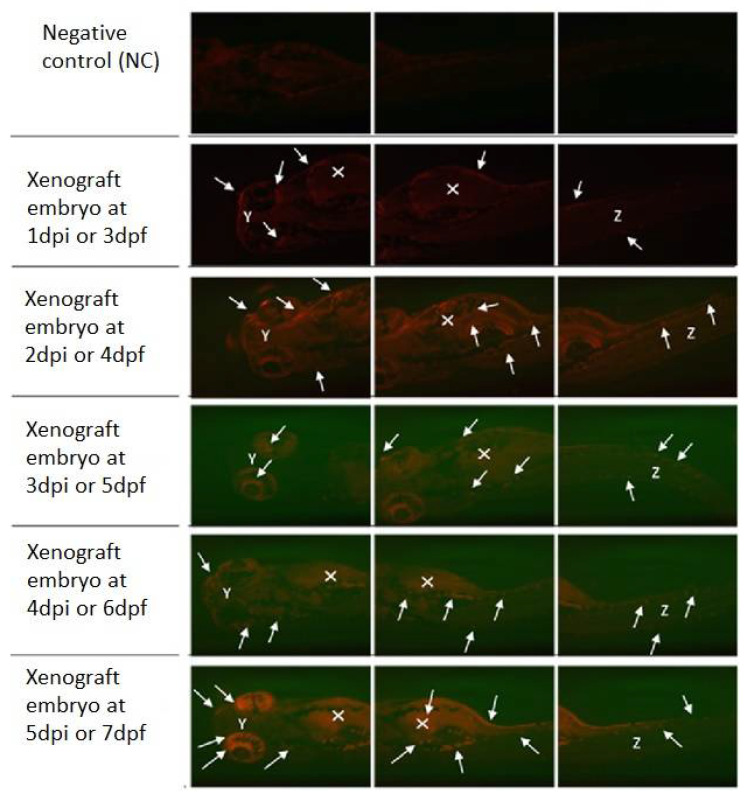
Zebrafish Xenograft model injected at 2 dpf. Representative fluorescence images of zebrafish screening at 3 dpf to 7 dpf using fluorescent microscopy and investigation of fluorescent K562 cells proliferation (white solid arrows) throughout the animal body (Y—eyes; X—yolk sac; Z—tail) using mCherry fluorescence filter. Original magnification 100×.

K562 CML cell line was also successfully transplanted when injected into the 48 hpf zebrafish embryos. Figure 8 represents a xenografted embryo from 1 day-post-injection until 5 days-post-injection (dpi) compared to a negative control embryo to differentiate between the autofluorescence of embryos. The fluorescently labeled cancer cells proliferated and resulted in an increase in tumor size and migration to distal sites of the embryo over time.

### 3.7. Xenograft Model Exposed to Loaded PLGA-PEG-PLGA NPs

**Figure 9:**
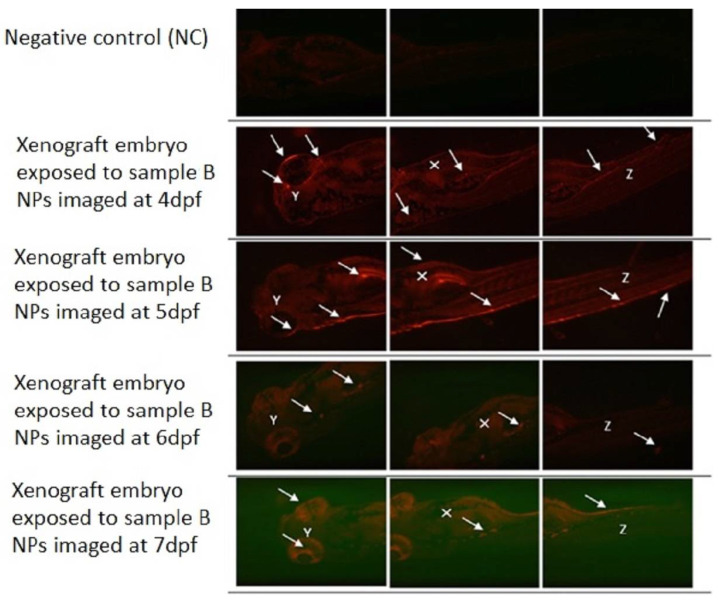
Xenograft model exposed sample B PLGA-PEG-PLGA NPs with 10 mg Ponatinib. Representative fluorescence images of zebrafish screening at 4 dpf until 7 dpf using fluorescent microscopy and investigation of fluorescent K562 cells proliferation (White solid arrows) throughout the animal body (Y—eyes; X—yolk sac; Z—tail) using mCherry fluorescence filter after exposing zebrafish embryos to of sample B PLGA-PEG-PLGA NPs (0.001 mg/mL). Original magnification 100×.

On the same day of K562 cells injection, the 2 dpf xenograft embryos were exposed to PLGA-PEG-PLGA NPs (0.001 mg/mL) loaded with 10 mg Ponatinib. Figure 9 demonstrates the effect of exposing xenograft embryos to sample B NPs (0.001 mg/mL) from day 2 post-injection until day 5 post-injection (dpi) compared to a negative control embryo.

**Figure 10:**
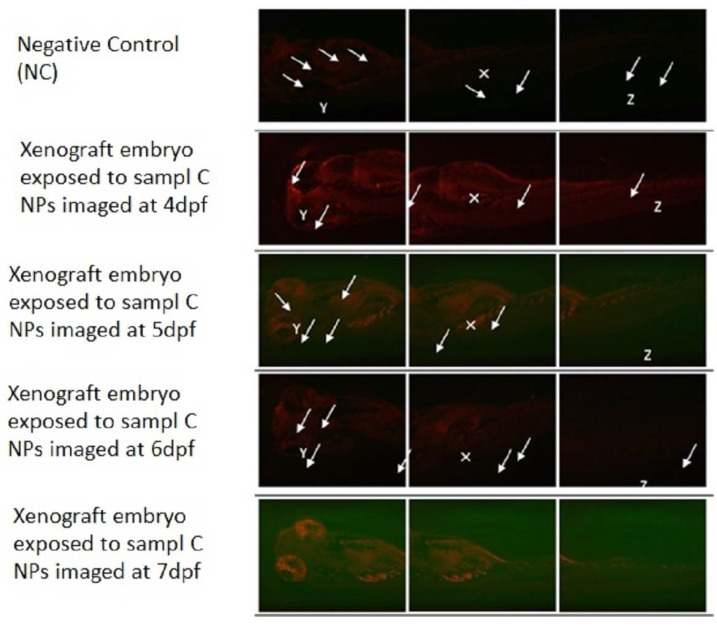
Xenograft model exposed to sample C PLGA-PEG-PLGA NPs. Representative fluorescence images of zebrafish screening at 4 dpf to 7 dpf using fluorescent microscopy and investigation of fluorescent K562 cells proliferation (white solid arrows) throughout the animal body (Y—eyes; X—yolk sac; Z—tail) using mCherry fluorescence filter after treating zebrafish embryos with sample C PLGA-PEG-PLGA NPs (0.001 mg/mL). Original magnification 100×.

As shown in Figure 10 for the group of xenografted 2 dpf embryos which were treated with sample C PLGA-PEG-PLGA NPs (0.001 mg/mL). The fluorescently labeled K562 cells increased in number and migrated to the distal location from the original site of injection over time as indicated by the white arrows. The yolk sac area (white X) (injection site of the K562 cells) showed the largest tumor cells mass. K562 cells had also circulated through the blood as shown in the embryo’s eyes (white Y) and tail (white Z). As seen in all Figure 8, Figure 9 and Figure 10, the loaded NPs took a long time to release Ponatinib, with no obvious decrease in tumor size.

### 3.8. The Uptake of Drug-Loaded PLGA-PEG-PLGA NPs

**Figure 11:**
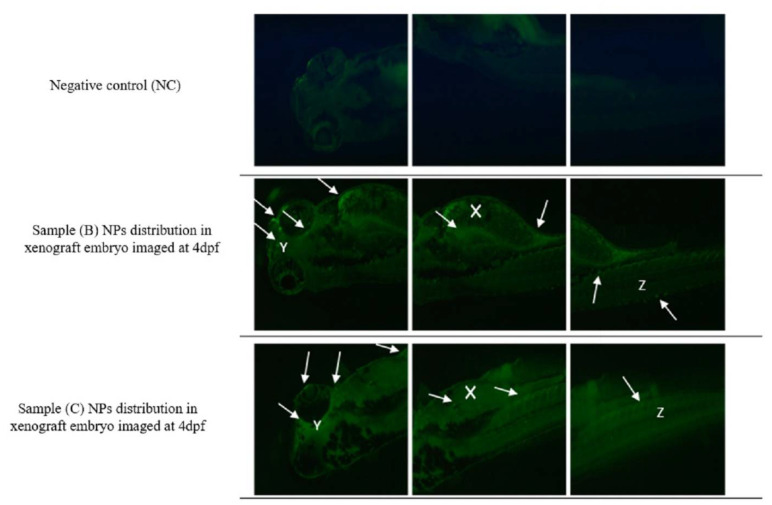
Loaded PLGA-PEG-PLGA NPs uptake. Representative fluorescence images of zebrafish screening at 4 dpf using fluorescent microscopy and investigation of fluorescent PLGA-PEG-PLGA NPs distribution (white solid arrows) throughout the animal body (Y—eyes; X—yolk sac; Z—tail) using GFP fluorescence filter. Original magnification 100×.

As shown in Figure 11 the sample B and C PLGA-PEG-PLGA NPs were successfully uptaken after 2 dpi, and NPs were stained using 5DTAF green fluorescent dye. White arrows are showing the fluorescence-labeled NPs through the embryo’s eye (white Y), yolk sac (white X), and tail (white Z).

## 4. Discussion

Since cancer is one of the main causes of death worldwide, many research studies are currently focusing on finding novel and efficient therapeutic tools to reduce side effects associated with conventional therapies for cancer [33]. Nanomedicine is one of the new approaches that overcome some of the related issues of conventional cancer therapies including their low bioavailability and low specificity. [34]. Thus, encapsulating the anti-cancer drugs or related active agents in NPs would increase their biocompatibility, solubility, stability in body fluids, and their retention time in tumor vasculature which would enhance the efficacy of the treatment [35,36,37].

Moreover, nanomedicine could also support the cardio-oncology field which is an inter-disciplinary field of studying, detecting, and treating cardiovascular adverse effects associated with cancer therapies [38]. Although TKIs are the effective and preferred choice of therapy in several types of cancers including CML, their toxicity remains a major concern, particularly their cardiotoxic effects in cancer patients [39]. This prompted us to investigate the efficacy of loading the Ponatinib drug as a member of the TKI family into the PLGA-PEG-PLGA NPs in the enhancement of the anti-cancer activity and the reduction of cardiotoxic effects related to this.

In the current study, the smart PLGA-PEG-PLGA NPs were synthesized by the nanoprecipitation method. The PLGA-PEG-PLGA NPs characteristics (size, shape, efficacy of loading, drug content, surface charge, and dissolution rate) had been investigated. The size of the unloaded PLGA-PEG-PLGA NPs was approximately 84.33 nm. Sulaiman et al. (2019) has shown that the PLGA-PEG-PLGA NPs size is in the range of 206 to 402 nm, Dimchevska et al., (2017) demonstrated that the size will vary depending upon the experimental conditions and polymer characteristics, and the most efficient way to optimize the formulation is to use experimental design for preparing PLGA-PEG-PLGA NPs [30,40]. SEM has shown that NPs are spherical in three dimensions with a smooth surface. This is because SEM is used to examine material surfaces and is based on scattered electrons [41]. While TEM showed that NPs are round in shape, TEM was used to show the NPs in a higher magnification as it is based on transmitted electrons; also, it has higher electron energy than SEM, which allows them to penetrate through the particles to define any internal elements in the particles [41]. The surface charge of the empty PLGA-PEG-PLGA NPs has been determined by measuring the Zeta potential that would aid in determining more about the particle properties and their interaction with the biological system. The unloaded PLGA-PEG-PLGA NPs revealed a negatively charged surface (−2.66 mV), as the polymer is affected by the PLGA copolymer end-group [40].

The cardiotoxicity of Ponatinib (2.5 μM) and the unloaded PLGA-PEG-PLGA NPs have been investigated to determine if this type of polymers would cause any cardiotoxicity or adverse effects. Our results demonstrated a significant effect of Ponatinib on reducing the velocity of aortic and PCV blood flow; these results came in agreement with a previous study conducted by Singh and coworkers [42]. The zebrafish model has been used in this study due to the transparency of the zebrafish embryos bodies which allows a non-invasive examination of the organ development [43]. The high concentrations of PLGA-PEG-PLGA NPs inside the studied model (1 mg/mL and 0.75 mg/mL) showed some toxicity as confirmed by the low survival rate, decreased heartbeat, low DA diameter, and decreased blood flow velocity of the treated groups when compared to the control group l. While the other groups treated with lower concentrations (0.5, 0.25, 0.1, and 0.05 mg/mL) demonstrated no significant difference in the survival rate and other aforementioned measured parameters when compared to the control group. This supports the fact that PLGA-PEG-PLGA NPs have low toxic effects and may be used to improve bioavailability and antitumor targeting [44,45,46].

Before Ponatinib loading to NPs cardiotoxicity experiments we investigated the effect of treating zebrafish embryos with different concentrations of Ponatinib on their viability, tail flicking, and hatching rate at different time points, it has been shown that Ponatinib had the minimum effect on all measured parameters at a concentration of (2.5 μM). The dosing rationale that was tested for Ponatinib was based on the literature [47]. Increased concentrations of Ponatinib significantly exerted a neuro/muscular toxic effect on embryos at 24 hpf reflected by the reduced tail flicking, these results can be explained by the toxic effect of Ponatinib at higher doses on angiogenesis as an important step in embryo organs development [48].

The smart NPs PLGA-PEG-PLGA loaded with Ponatinib (samples A—3%, B—6.05%, and C—10.5% drug loading) were used to reduce the drug’s toxic side effects, especially on the cardiovascular system. The loading of the drug was successfully performed, and this was indicated by the change in the surface charge of the PLGA-PEG-PLGA NPs from negative (−2.66 mV) to positive (12.3, 15.2, and 16.7 mV) charge for C, B, and A samples, respectively during the drug incorporation. Ponatinib surface charge is positive (30.86 mV) due to protonation of its on its terminal methylpiperazinyl nitrogen across water [49]. This phenomenon is often seen in nanoparticles. One example is described by Ku et al. (2010), who disclosed the change in the FMSNs surface charge from negative (−22.43 mV) to positive (18.93 mV) due to the conjugation of PAMAM of a positive charge, and eventually, the charged change almost to neutral (1.49 mV) revealing an additional modification of PEG [50]. HPLC analysis has confirmed the presence of Ponatinib in the PLGA-PEG-PLGA NPs with drug content of 3% for sample A, 6.05% for sample B, and 10.5% for sample C. Increasing concentrations of Ponatinib during the NPs preparation resulted in increased efficacy of encapsulation, probably due to electrostatic interaction between the protonated drug and the hydroxyl-terminated polymer which improved packaging and chain entanglement during the nanoprecipitation. For sample A, prepared using the lowest quantity of Ponatinib (5 mg; 1:6 drug/polymer ratio), resulting in the lowest efficacy of encapsulation and drug content, there was an initial burst release of the drug within a short time after the immersion in the dissolution medium which is additional confirmation of improved packaging of the drug and polymer chains with a higher concentration of Ponatinib during the sample preparation. Burst release is undesirable, as it would shorten the drug’s overall therapeutic duration and increase the drug’s toxic potential due to excessive burst release [51]. on the other hand, PLGA-PEG-PLGA NPs prepared with higher concentrations of Ponatinib during the nanoprecipitation process (10mg Ponatinib, a drug-to-polymer ratio of 1:3 for sample B; and 15 mg Ponatinib, a drug-to-polymer ratio of 1:2 for sample C) did not show the same pattern of burst release over a 48 h period, marking these concentrations and samples out to be better candidates for in vivo testing.

The cardiotoxicity of different concentrations of sample B and sample C PLGA-PEG-PLGA NPs was tested in zebrafish embryos before the investigation of their efficacy, as a less cardiotoxic therapeutic tool to treat CML. The concentration of 1, 0.75, 0.5, 0.25, 0.1, 0.025, 0.05, and 0.01 mg/mL NPs of all groups (samples A, B, and C) had shown very clear toxicity based on demonstrated embryo survival rates which were the lowest associated with abnormal morphology.This was similar to the toxicity results of the treatment of Ponatinib drug only, as the embryos were deformed with heart edema and abnormal heart structure as well as for the absence of blood flow in the PCV and DA. This could be due to the burst release of Ponatinib which has been determined to cause cardiotoxicity. However, the lowest concentrations (0.005 and 0.0025 mg/mL) had shown a better effect but still, there were some observed abnormalities in the embryos, thus sample A PLGA-PEG-PLGA NPs were excluded and a lower concentration (0.001 mg/mL) from samples B and C PLGA-PEG-PLGA NPs were tested, and they demonstrated the best results in terms of survival rate, normal morphology, and cardiac output.

Successfully, a zebrafish xenograft model has been generated to investigate the efficacy of those loaded with sample B and C PLGA-PEG-PLGA NPs, in reducing cardiotoxicity and as effective anti-cancer therapy to treat CML. This was achieved by transplanting the human K562 cell line into 2 dpf zebrafish embryos. This xenograft model has also been successfully generated and confirmed by the spread of the tumor cells to distal sites from the yolk sac throughout 6 days-post-injection, which is consistent with previous studies. Corkery et.al. (2011) has also used the K562 cells that were stained by the CM-Dil dye to give a red fluorescence color. These cells were then transplanted into the zebrafish embryos and the embryos were then kept for 1 h at 28 °C for a recovery period and this aided in enhancing the embryos’ survival rate [52]. However, in this study, the embryos have been immediately incubated at 34 °C without a recovery period and this might be the reason behind the low survival rate of the injected embryos after 1 day-post-injection. Moreover, Pruvot et al. (2011) has shown successful transplantation of the K562 cell line into the zebrafish embryos [53].

Finally, samples B and C PLGA-PEG-PLGA NPs (0.001 mg/mL) concentration were introduced to the injected zebrafish embryos (2 dpf) half an hour after the injection. The tumor cells are not reduced clearly over the 6 days after injection, this could be due to the long release time of Ponatinib from the PLGA-PEG-PLGA NPs.

Possible limitations of this study includes that it being mostly dependent on zebrafish embryos that needed proper care and training for handling and that the xenograft model required an even a higher level of handling as the embryos are injured. The treated groups of the zebrafish embryos with the loaded PLGA-PEG-PLGA NPs were only observed till the ethical endpoint of 7-dpf,;thus, the effect of the loaded PLGA-PEG-PLGA NPs in reducing tumor cells was only observed for a few days despite the fact that Ponatinib release that could happen after a few days of the endpoint. For that, xenografted embryos need to be observed for a longer time, e.g., 10-dpf. Moreover, due to the lack of FTIR < X-ray and DSC studies, the paradox of the presence of burst release at the lowest drug concentration cannot be explained. Moreover, deducting background fluorescence per unit area of the fluorescence images would give better quantitative measurements.

## 5. Conclusions

In summary, the zebrafish is a suitable animal model for investigating the cardiotoxicity associated with anti-cancer drugs such as TKIs, determining the optimum concentration of smart NPs with the least side effects, and generating a xenograft model of several cancer types.

In this study, PLGA-PEG-PLGA NPs were synthesized to carry the TKIs drugs. These NPs have been shown to carry Ponatinib drugs (Samples B and C for a long time, allowing for longer circulation in the zebrafish body. Zebrafish animal model was used for testing the cardiotoxicity of a range of different concentrations of loaded and unloaded PLGA-PEG-PLGA NPs and the least concentrations were shown to be of low toxicity and enhanced survival rate. The concentrations of 0.1 and 0.05 mg/mL of the unloaded PLGA-PEG-PLGA NPs are the best in terms of low cardiotoxicity and high survival rate, while 0.001 mg/mL concentration of samples B or C PLGA-PEG-PLGA NPs has been shown to be the optimum concentration among the rest of the concentrations. Lastly, these loaded NPs have been exposed to the successfully generated CML xenograft zebrafish model, however, no obvious reduction in the tumor mass was seen, indicating the slow release of Ponatinib from PLGA-PEG-PLGA NPs.

Generally, PLGA-PEG-PLGA NPs could be a good candidate for CML treatment, but their cellular internalization should be enhanced. This could be achieved by coating and labeling the surface of PLGA-PEG-PLGA NPs with specific ligands that are unique to CML cells.

## Figures and Tables

**Table 1:** Unloaded PLGA-PEG-PLGA NPs surface charge.

Sample Name	Zeta Potential (mV)
Unloaded Nano particles 1	−2.48
Unloaded Nano particles 2	−2.85
Unloaded Nano particles 3	−2.65
Mean	−2.66
STD	0.185

**Table 2:** Ponatinib drug surface charge.

Sample Name	Zeta Potential (mV)
Ponatinib 1	32.5
Ponatinib 2	29.7
Ponatinib 3	33.7
Ponatinib 4	31.7
Ponatinib 5	26.7
Mean	30.86
STD	2.744

## Supplementary Materials

The following supporting information can be downloaded at: https://www.mdpi.com/article/10.3390/ma15113960/s1. Figure S1: QU-IACUC approval; Figure S2: Representative fluorescence images for K562 cells stained with CM-Dil dye (Red). Fluorescently labeled K562 cells at magnification 60×; Scale bar, 0.03 mm; Figure S3: TEM and SEM micrographs of PLGA-PEG-PLGA NPs. (A) TEM image of PLGA-PEG-PLGA Np on scale bar, 50 nm. (B) SEM image of PLGA-PEG-PLGA NPS on scale bar, 1 µm; Figure S4: Ponatinib loaded PLGA-PEG-PLGA NPs intensity-based particles size distribution. Representative graphs of Nanosizer 2000-Malvern for loaded PLGA-PEG-PLGA NPs size. (A) The size of Sample A PLGA-PEG-PLGA NPs is 74.55+/−28.74 (d.nm) ± SD. (B) The size of Sample B PLGA-PEG-PLGA NPs is 125+/−26.91 (d.nm) ± SD. (C) The size of Sample C PLGA-PEG-PLGA NPs is 116.9+/−42.92 (d.nm) ± SD; Figure S5: Unloaded PLGA-PEG-PLGA NPs intensity-based particles size distribution. Representative graph of Nanosizer 2000-Malvern for unloaded PLGA-PEG-PLGA NPs size. The size of unloaded PLGA-PEG-PLGA NPs is 84.33+/−13.83 (d.nm) ± SD. Figure S6: Ponatinib Dissolution Rate from PLGA-PEG-PLGA NPs. Representative graphs of HPLC for Ponatinib Dissolution Rate from PLGA-PEG-PLGA NPs (A) Standard graph of Ponatinib drug peak at 1.678 RT. (B) sample A PLGA-PEG-PLGA NPs at 1 h. (C) sample A PLGA-PEG-PLGA NPs at 3 h. (D) sample A PLGA-PEG-PLGA NPs at 5 h. (E) sample A PLGA-PEG-PLGA NPs at 24 h. (F) sample B PLGA-PEG-PLGA NPs at 1 h. (G) PLGA-PEG-PLGA NPs at 3 h. (H) sample B PLGA-PEG-PLGA NPs at 5 h. (I) sample B PLGA-PEG-PLGA NPs at 24 h. (J) sample C PLGA-PEG-PLGA NPs at 1 h. (K) sample C PLGA-PEG-PLGA NPs at 3 h. (L) sample C PLGA-PEG-PLGA NPs at 5 h. (M) PLGA-PEG-PLGA NPs at 24 h. (N) sample B PLGA-PEG-PLGA NPs at 48 h. (O) sample C PLGA-PEG-PLGA NPs at 48 h.
