# Supplementary material for: Reduced Cardiotoxicity of Ponatinib-Loaded PLGA-PEG-PLGA Nanoparticles in Zebrafish Xenograft Model
Source: Materials (Basel). 2022 Jun 2;15(11):3960. doi: 10.3390/ma15113960 (PMC9182153; doi:10.3390/ma15113960)
Supplement: Supplementary file 1 [file materials-1691261-supplementary.pdf › materials-1691261-supplementary.pdf]

Reduced Cardiotoxicity of Ponatinib Loaded PLGA-PEG-PLGA Nanoparticles in Zebrafish Xenograft Model

جامعة قطر
QATAR UNIVERSITY

Institutional Animal Care & Use Committee (QU-IACUC)

QATAR UNIVERSITY, PO BOX 2713, DOHA, QATAR
TEL: +974 4403-6676 **FAX:** +974 4403-3901
Email: QU.IACUC@qu.edu.qa
Assurance Registration #: IACUC-QU-2019-002
Assurance #: IACUC-A-QU-2019-0004

January 7th, 2021

TO: **Dr. Huseyin Yalcin,** Biomedical Research Center, Qatar University
Email: hyalcin@qu.edu.qa

FROM: Qatar University - Institutional Animal Care & Use Committee (QU-IACUC)

SUBJECT: **Letter of IACUC Protocol Approval (QU-IACUC 019/2020) – Designated Member Review (DMR)**
Project Title: "Development and in vivo testing of smart nanoparticles for enhanced anti-cancer activity and reduced cardiotoxicity associated with Tyrosine kinase inhibitors"

Grant: QUST-2-CHS-2020-11

Dear Dr. Huseyin,

The Qatar University Institutional Animal Care and Use Committee (QU-IACUC) has **APPROVED** by the designated member review the above referenced Animal Use Protocol # **QU-IACUC 019/2020.**

Date of Initial Approval: January 7, 2021 **Date of Expiration:** January 6, 2022

The IACUC staff will make every effort to send the Principal Investigator annual reminders. However, it is the Principal Investigator's sole responsibility to submit needed renewals and Annual Review **at least one month in advance** of the annual review due dates to ensure continuing IACUC approval. **It is very important that these deadlines are not missed**. Failure to submit an Annual Review/Renewal on time will result in all persons listed under this protocol losing access to zebrafish facility and animal ordering and may potentially result in the termination of the protocol. QU-IACUC deals with expired protocols approvals as new submissions.

Please note that to continue this research/protocol beyond the **three-year period since its start**, a new protocol submission will be required. To avoid a lapse in IACUC approval, it is essential that the completed annual renewal protocol be submitted and approved by the IACUC prior to its expiration date.

Ministry of Public Health regulations do not permit the IACUC to extend any approval periods. If a renewal protocol has not been processed and approved by the IACUC prior to its expiry, IACUC approval for the work under the above referenced protocol will expire. Should IACUC approval expire, all activities involving the care and use of animals must cease immediately. Any activities conducted under the protocol after expiration will be in direct violation of governmental regulations and institutional/IACUC policies.

It is the responsibility of the Principal Investigator to notify the IACUC of any proposed changes regarding the work described within this protocol. The Principal Investigators listed above agree that no such changes will be implemented until approved by the IACUC, except where absolutely necessary to eliminate apparent immediate hazards to person(s) and/or animal(s).

1

جامعة قطر
QATAR UNIVERSITY

Kindly refer to the above Animal Use Protocol number in all of your future correspondence with us pertaining to this project.

Sincerely,

Dr. Abdelali Agouni, MSc, PhD, FHEA
Chair of QU-IACUC
Qatar University
Doha, Qatar

جامعة قطر
QATAR UNIVERSITY
IACUC
Institutional Animal Care and Use Committee

copy: QU-IACUC files

2

Figure S1. QU-IACUC approval

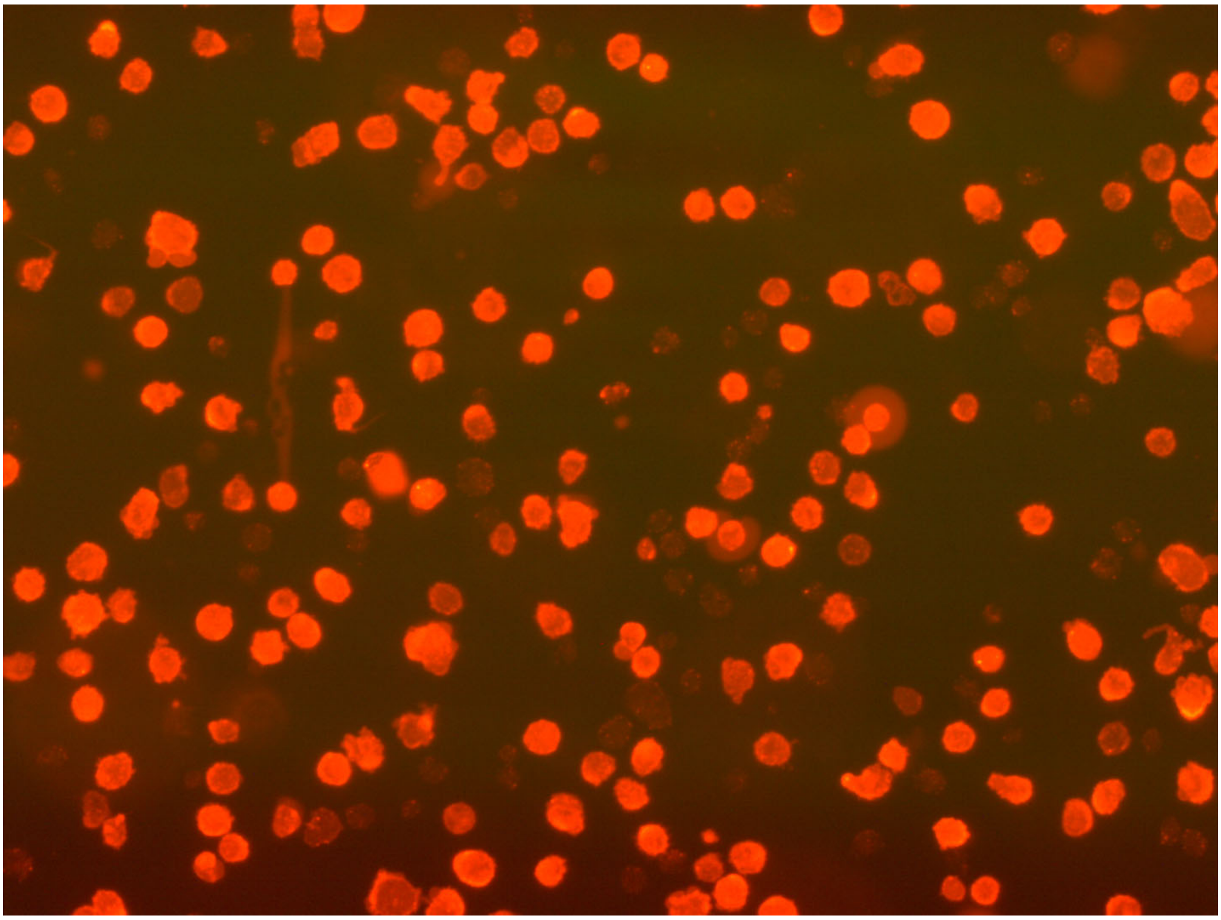

Figure S2. Fluorescently labelled K562. Representative fluorescence images for K562 cells stained with CM-Dil dye (Red). Fluorescently labeled K562 cells at magnification 60X

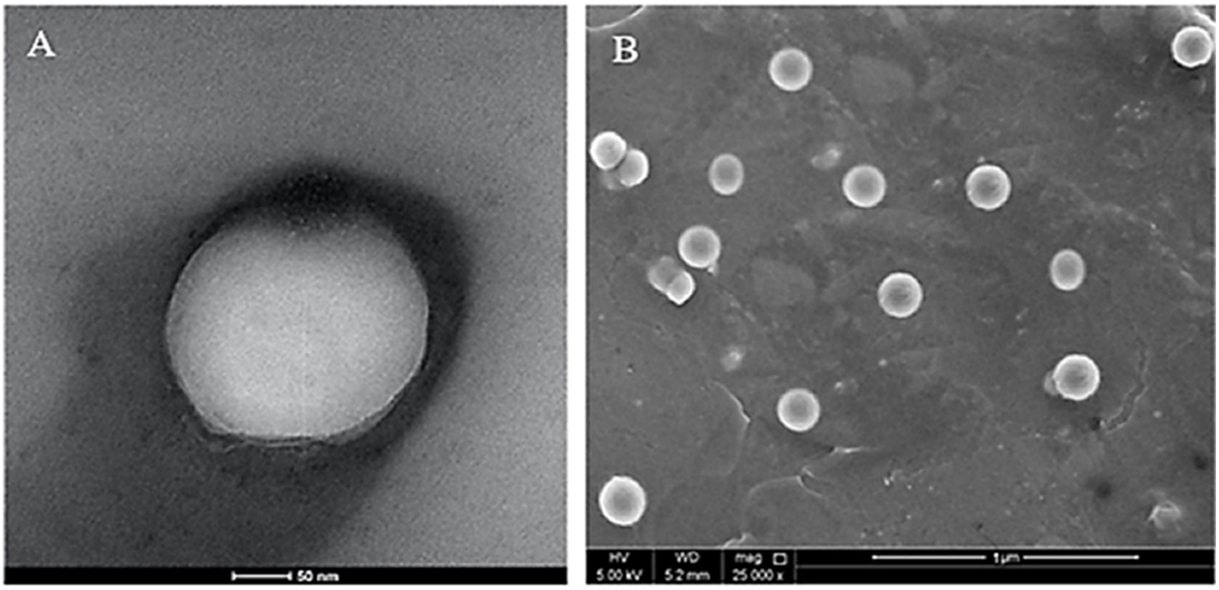

Figure S3. TEM and SEM micrographs of PLGA-PEG-PLGA NPs.

(A) TEM image of PLGA-PEG-PLGA Np on scale bar, 50 nm. **(B)** SEM image of PLGA-PEG-PLGA Nps on scale bar, 1µm.

A

			Size (d.nm):	% Intensity:	St Dev (d.nm):
Z-Average (d.nm):	74.55	Peak 1:	73.61	100.0	28.74
PdI:	0.259	Peak 2:	0.000	0.0	0.000
Intercept:	0.882	Peak 3:	0.000	0.0	0.000
Result quality :	Good				

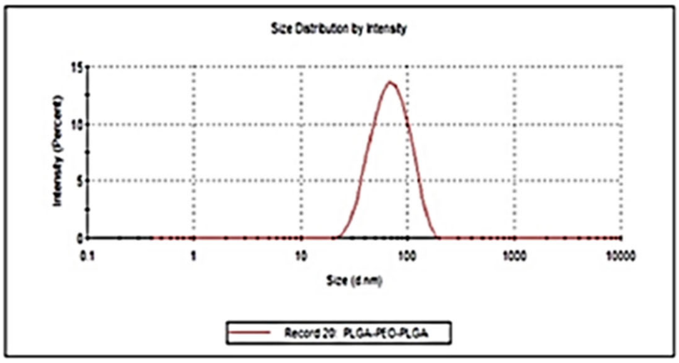

B

			Size (d.nm):	% Intensity:	St Dev (d.nm):
Z-Average (d.nm):	125.0	Peak 1:	97.42	100.0	26.91
PdI:	0.267	Peak 2:	0.000	0.0	0.000
Intercept:	0.951	Peak 3:	0.000	0.0	0.000
Result quality :	Good				

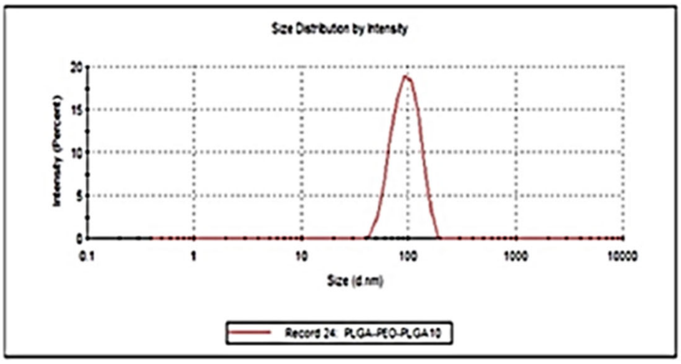

C

			Size (d.nm):	% Intensity:	St Dev (d.nm):
Z-Average (d.nm):	116.9	Peak 1:	117.5	96.1	42.92
PdI:	0.297	Peak 2:	31.39	3.9	5.020
Intercept:	0.945	Peak 3:	0.000	0.0	0.000
Result quality :	Good				

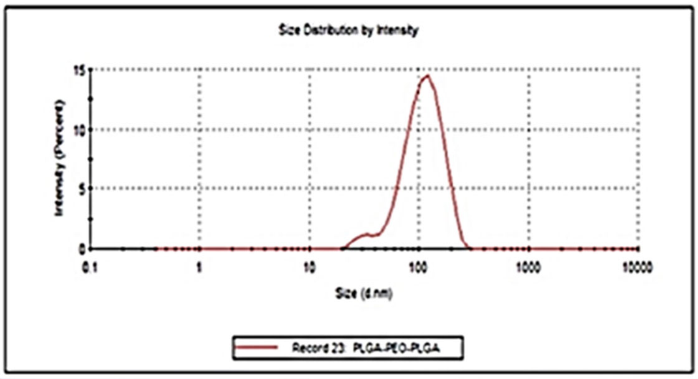

A

			Size (d.nm):	% Intensity:	St Dev (d.nm):
Z-Average (d.nm):	74.55	Peak 1:	73.61	100.0	28.74
PdI:	0.259	Peak 2:	0.000	0.0	0.000
Intercept:	0.882	Peak 3:	0.000	0.0	0.000
Result quality :	Good				

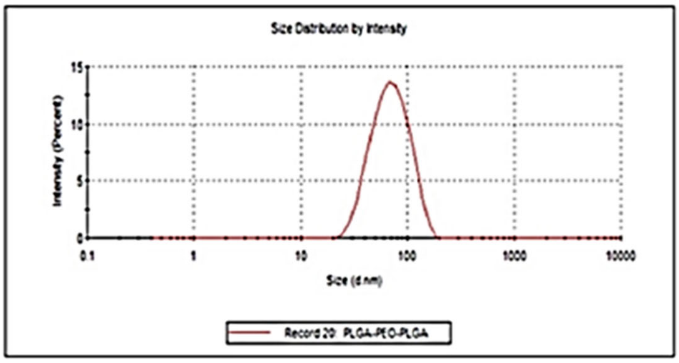

B

			Size (d.nm):	% Intensity:	St Dev (d.nm):
Z-Average (d.nm):	125.0	Peak 1:	97.42	100.0	26.91
PdI:	0.267	Peak 2:	0.000	0.0	0.000
Intercept:	0.951	Peak 3:	0.000	0.0	0.000
Result quality :	Good				

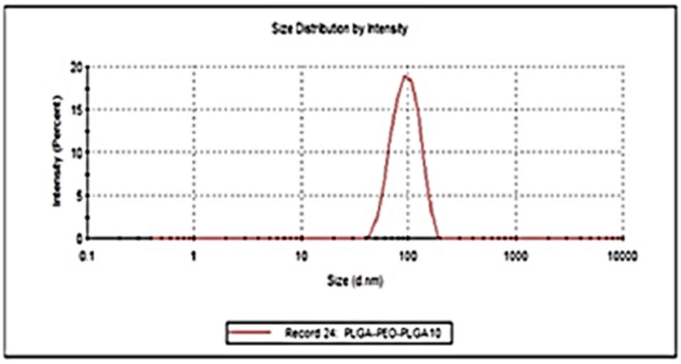

C

			Size (d.nm):	% Intensity:	St Dev (d.nm):
Z-Average (d.nm):	116.9	Peak 1:	117.5	96.1	42.92
PdI:	0.297	Peak 2:	31.39	3.9	5.020
Intercept:	0.945	Peak 3:	0.000	0.0	0.000
Result quality :	Good				

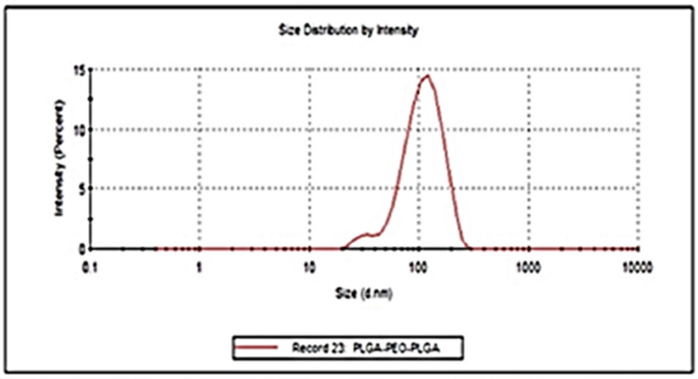

Figure S4. Ponatinib loaded PLGA-PEG-PLGA NPs intensity-based particles size distribution.

Representative graphs of Nanosizer 2000-Malvern for loaded PLGA-PEG-PLGA NPs size **(A)** The size of Sample A PLGA-PEG-PLGA NPs is 74.55+/- 28.74 (d.nm)±SD **(B)** The size of Sample B PLGA-PEG-PLGA NPs is 125+/- 26.91 (d.nm)±SD **(C)** The size of Sample C PLGA-PEG-PLGA NPs is 116.9+/- 42.92 (d.nm)±SD.

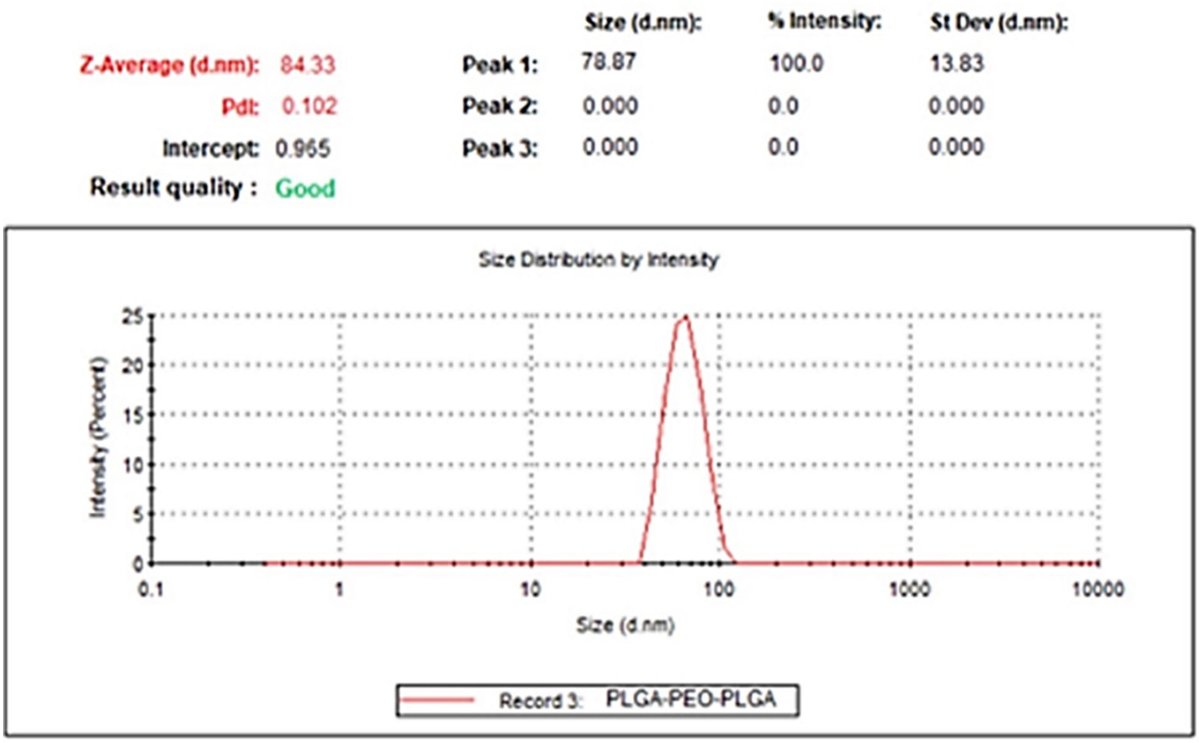

Figure S5. Unloaded PLGA-PEG-PLGA NPs intensity based particles size distribution. Representative graph of Nanosizer 2000-Malvern for unloaded PLGA-PEG-PLGA NPs size. Size of unloaded PLGA-PEG-PLGA NPs is 84.33+/-13.83 (d.nm)±SD.

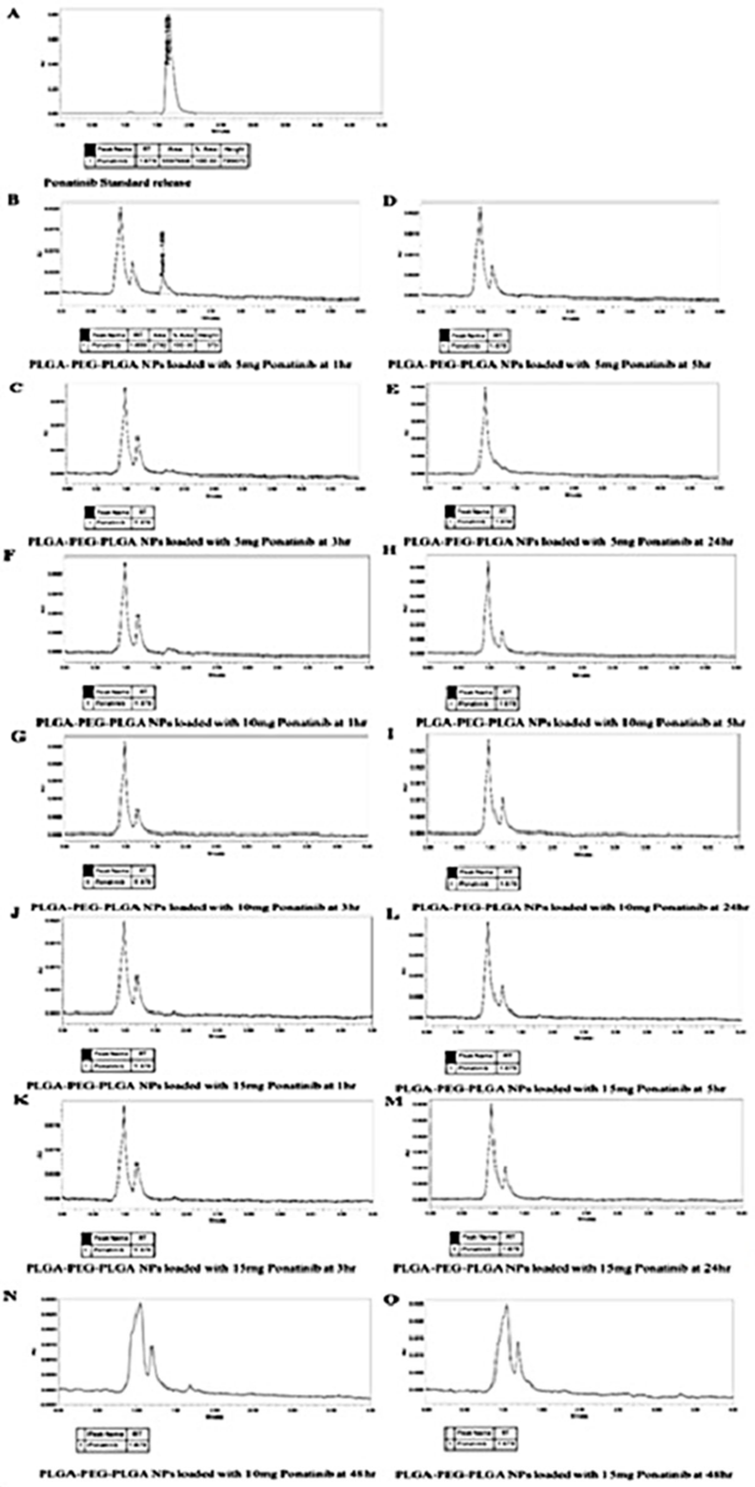
A
Ponatinib Standard release
B
PLGA-PEG-PLGA NPs loaded with 5mg Ponatinib at 1hr
D
PLGA-PEG-PLGA NPs loaded with 5mg Ponatinib at 5hr
C
PLGA-PEG-PLGA NPs loaded with 5mg Ponatinib at 3hr
E
PLGA-PEG-PLGA NPs loaded with 5mg Ponatinib at 24hr
F
PLGA-PEG-PLGA NPs loaded with 10mg Ponatinib at 1hr
H
PLGA-PEG-PLGA NPs loaded with 10mg Ponatinib at 5hr
G
PLGA-PEG-PLGA NPs loaded with 10mg Ponatinib at 3hr
I
PLGA-PEG-PLGA NPs loaded with 10mg Ponatinib at 24hr
J
PLGA-PEG-PLGA NPs loaded with 15mg Ponatinib at 1hr
L
PLGA-PEG-PLGA NPs loaded with 15mg Ponatinib at 5hr
K
PLGA-PEG-PLGA NPs loaded with 15mg Ponatinib at 3hr
M
PLGA-PEG-PLGA NPs loaded with 15mg Ponatinib at 24hr
N
PLGA-PEG-PLGA NPs loaded with 10mg Ponatinib at 48hr
O
PLGA-PEG-PLGA NPs loaded with 15mg Ponatinib at 48hr

Figure S6. Ponatinib Dissolution Rate from PLGA-PEG-PLGA NPs.

Representative graphs of HPLC for Ponatinib Dissolution Rate from PLGA-PEG-PLGA NPs **(A)** Standard graph of Ponatinib drug peak at 1.678 RT. **(B) sample A** PLGA-PEG-PLGA NPs at 1hr. **(C) sample A** PLGA-PEG-PLGA NPs at 3hr. **(D) sample A** PLGA-PEG-PLGA NPs at 5hr. **(E) sample A** PLGA-PEG-PLGA NPs at 24hr. **(F) sample B** PLGA-PEG-PLGA NPs at 1hr. **(G)** PLGA-PEG-PLGA NPs at 3hr. **(H) sample B** PLGA-PEG-PLGA NPs at 5hr. **(I) sample B** PLGA-PEG-PLGA NPs at 24hr. **(J) sample C** PLGA-PEG-PLGA NPs at 1hr. **(K) sample C** PLGA-PEG-PLGA NPs at 3hr. **(L) sample C** PLGA-PEG-PLGA NPs at 5hr. **(M)** PLGA-PEG-PLGA NPs at 24hr. **(N) sample B** PLGA-PEG-PLGA NPs at 48hr. **(O) sample C** PLGA-PEG-PLGA NPs at 48hr.
